# Defining Nutrient Combinations for Optimal Growth and Polyhydroxybutyrate Production by *Methylosinus trichosporium* OB3b Using Response Surface Methodology

**DOI:** 10.3389/fmicb.2018.01513

**Published:** 2018-07-18

**Authors:** Jorge A. Zaldívar Carrillo, Lisa Y. Stein, Dominic Sauvageau

**Affiliations:** ^1^Department of Chemical and Materials Engineering, University of Alberta, Edmonton, AB, Canada; ^2^Department of Biological Sciences, University of Alberta, Edmonton, AB, Canada

**Keywords:** methanotroph growth, *Methylosinus trichosporium*, polyhydroxybutyrate (PHB), optimization of biopolymer production, response surface methodology

## Abstract

Methane and methanol are common industrial by-products that can be used as feedstocks for the production of value-added products by methylotrophic bacteria. Alphaproteobacterial methanotrophs are known to produce and accumulate the biopolymer polyhydroxybutyrate (PHB) under conditions of nutrient starvation. The present study determined optimal production of biomass and PHB by *Methylosinus trichosporium* OB3b as a function of carbon source (methane or methanol), nitrogen source (ammonium or nitrate), and nitrogen-to-carbon ratio during growth. Statistical regression analysis with interactions was performed to assess the importance of each factor, and their respective interactions, on biomass and PHB production. Higher biomass concentrations were obtained with methane as carbon source and with ammonium as nitrogen source. The nitrogen source that favored PHB production was ammonium for methane-grown cells and nitrate for methanol-grown cells. Response surface methodology (RSM) was used to determine conditions leading to optimal biomass and PHB production. As an example, the optimal PHB concentration was predicted to occur when a mixture of 30% methane and 70% methanol (molar basis) was used as carbon source with nitrate as nitrogen source and a nitrogen-to-carbon molar ratio of 0.017. This was confirmed experimentally, with a PHB concentration of 48.7 ± 8.3 mg/L culture, corresponding to a cell content of 52.5 ± 6.3% (cell dry weight basis). Using RSM to simultaneously interrogate multiple variables toward optimized growth and production of biopolymer serves as a guide for establishing more efficient industrial conditions to convert single-carbon feedstocks into value-added products.

## Introduction

The impact of plastic pollution has motivated interest in economically viable biodegradable polymers. One such class of biopolymers is polyhydroxyalkanoates (PHAs), of which polyhydroxybutyrate (PHB) is the most widely studied representative. Current industrial production relies on using sugars as feedstock, which translates into high production costs and competition with food production. A promising alternative involves using methanotrophic bacteria that convert single-carbon compounds into PHB (Chidambarampadmavathy et al., 2017). The use of residual methane and methanol, both low value common industrial by-products, reduces both the costs of production of biomolecules, such as PHB, compared to sugar-based feedstocks (Fei et al., 2014; Strong et al., 2015) and carbon emissions from the source industry.

PHB production by methanotrophic bacteria generally requires a starvation signal that is usually accomplished by providing excess carbon while limiting the nitrogen source (Lee, 1996; Pieja et al., 2011; Zhang et al., 2017). Alphaproteobacterial methanotrophs utilize the serine cycle for formaldehyde assimilation and feed acetyl-CoA from this cycle into PHB biosynthesis (Pieja et al., 2011). Although central carbon metabolism is essentially identical among the alphaproteobacterial methanotrophs, there is substantial strain-to-strain variability in terms of preferred N-source and engagement of PHB production during N-starvation. For instance, *Methylosinus trichosporium* OB3b cultivated in medium with nitrate produced more PHB upon N-limitation than cultures grown with ammonium, whereas the opposite result was observed with *Methylocystis parvus* OBBP (Rostkowski et al., 2013; Zhang et al., 2017). The physiological influence of N-source on PHB production in methanotrophs has not yet been resolved, let alone the impact of using a combination of N-sources, nor has strain-to-strain preference for one N-source over another. In addition, the production of PHB using methanol as a sole carbon and energy source, or a mixture of methane and methanol, instead of methane alone, has not been extensively investigated for methanotrophs.

The present study aims to optimize the production of biomass and PHB in *M. trichosporium* OB3b in relation to carbon source (methane or methanol), nitrogen source (nitrate or ammonium), and the carbon to nitrogen ratio using statistical regression analysis combined with response surface methodology (RSM). RSM is a collection of statistical tools that avoids the time consuming and expensive optimization of individual parameters required in full-factorial methodology to achieve a desired response. Instead, statistical regression analysis and RSM help determine the effects of multiple variables simultaneously (i.e., independent, or process, variables) toward the response of interest (i.e., response variable) and also measure the significance of each independent variable on the response (Myers et al., 2016). This analysis enabled us to determine optimal carbon and nitrogen combinations toward the highest yield of either biomass or PHB per liter of culture of *M. trichosporium* OB3b. This study also highlights the potential of RSM to optimize methanotrophic growth medium toward production of a valuable biopolymer despite the complex interacting effects of carbon and nitrogen sources on metabolism.

## Materials and Methods

### Cultivation

*Methylosinus trichosporium* OB3b is maintained in the Stein lab and was originally acquired as a gift from Dr. Alan DiSpirito, Iowa State University. Nitrate mineral salts (NMS) and ammonium mineral salts (AMS) media (Whittenbury et al., 1970) were used in this study. These media normally contain 10 g/L KNO_3_ or 5 g/L NH_4_Cl, respectively, but these concentrations were varied as required using 99 mM stock solutions of KNO_3_ or NH_4_Cl to achieve the desired nitrogen to carbon ratios tested in this study.

Cultures (100 mL) were grown in serum-capped Wheaton bottles (311 mL). Prior to inoculation, filter-sterilized (0.22 μm; Corning United States) methanol (Fisher Scientific) at 10 or 20 mM, filter-sterilized methane (95 + 5% CO_2_; Praxair, Canada) at 20 mmol/L of medium, or a mixture of the two carbon sources totaling 20 mmol carbon per liter of liquid was added as carbon source. Prior to the addition of methane, an equivalent amount of air was removed from the bottles to maintain the same headspace pressure as the methanol-amended cultures. Cultures were inoculated with 1–4% v/v of 2–5 day old stationary phase culture by injection through the septum. Cultures were incubated at 30°C and 150 rpm until analysis.

Cell dry weight was measured by extracting 20 to 30 mL culture and centrifuging at 10,000 ×*g* at 4°C for 10 min (Sorvall RC 6 Plus, SS-34 rotor; Thermo Scientific). The supernatant was discarded and the pellet was resuspended in 10 mL deionized water and transferred to a tared weigh dish. The dish was placed in an oven at 60°C for drying to constant weight. Optical density of cultures was measured at a wavelength of 540 nm using a spectrophotometer (Ultrospec 50, Biochrom). A calibration curve was prepared to convert OD measurements to unit cell dry weight.

### PHB Measurement

The quantification of PHB was performed via gas chromatography using a modified methodology (Braunegg et al., 1978; Oehmen et al., 2005). A 7–10 mL sample of culture was centrifuged at 2,988 ×*g* for 30 min. The supernatant was discarded and the pellet resuspended in a solution containing 2 mL chloroform and 2 mL of benzoic acid solution (40 mg/L) dissolved in methanol and acidified with 3% concentrated sulfuric acid. The sample was digested for 5 h – to depolymerize the polymer to its monomer and esterify it with methanol – in a capped glass vial in a boiling water bath. After cooling, 1 mL deionized water was added and the sample was vortexed and left to stand for phase separation. The organic phase was analyzed for methyl 3-hydroxybutyrate, the methylated form of the PHB monomer, using a gas chromatograph (7890A, Agilent Technologies) equipped with an autosampler (G4513A, Agilent Technologies) and fitted with a 30 m × 250 μm DB-5ms column (Agilent Technologies). The injector temperature was held at 250°C, and the oven temperature was held at 80°C for 1 min, raised to 120°C at a rate of 10°C/min, and then to 270°C at 30°C/min, before being held at that temperature for 3 min. 3-samples were injected at a split ratio of 1:10. A flame ionization detector (FID) at 300°C was used. Helium was used as the carrier gas at a flow of 1.5 mL/min. The peak of methyl 3-hydroxybutyrate was resolved at 2.8 min; an internal standard of methyl benzoate was resolved at 5.4 min (Supplementary Figure S7).

### Statistical Analysis

Analysis of variance (ANOVA) was used to determine significant effects of each variable and the combinations of variables on bacterial growth. Clustered Plot Analysis was used to visualize the ANOVA results and summarize the mean, median, standard deviation, and outliers for each group of compared data. Visualization used the Matlab script^1^ “notBoxPlot.m.”

The impacts of C-source, N-source, N:C ratio and history of the inoculum on growth was first investigated through statistical regression analysis with interactions. To do so, a 2^4^ full factorial experimental design was performed in which each of these four factors had two levels (**Table 1**). A total of 100-mL cultures in 250-mL Wheaton bottles were performed in triplicate and analyzed for each condition. Nitrogen concentrations of 10 or 1 mM were used to achieve the high and low nitrogen-to-carbon ratio, respectively. In addition, fresh (4–8 weeks) and aged (5.5–6.3 months) inocula were used, grown initially from either methane or methanol, with ammonium or nitrate as N-source, accordingly. A separate block of experiments was conducted in duplicate with methanol grown cultures with ammonium only, initiated from 2.1 to 2.3 week and 3.1–3.3 week old inocula. These additional experiments were included due to the extensive lag periods observed in experiments performed with methanol using the older inocula; they were treated separately in the analysis and modeling exercise.

**Table 1:** Factors and levels used in the second-order statistical regression analysis.

Factors investigated	Design levels	
Carbon source	Methane (CH_4_)	+1
	Methanol (CH_3_OH)	-1
Nitrogen source	Ammonium (NH_4_^+^)	+1
	Nitrate (NO_3_^-^)	-1
Nitrogen-to-Carbon ratio (N:C)	Low^1^	+1
	High^2^	-1
Inoculum history^3^	Fresh methane-grown	+1
	Aged methane-grown	-1
	Fresh methanol-grown	+1
	Aged methanol-grown	-1


*^1^Low level of N:C was 0.046 for methane and 0.1 for methanol. ^2^High level of N:C was 0.46 for methane and 1.0 for methanol. ^3^Inocula previously grown on methane with ammonium or nitrate were used to start both methane and methanol cultures; inocula grown on methanol with ammonium were used to start only methanol cultures.*

The RSM used a face-centered central composite design to depict the interactions among the variables in a cuboidal experimental space. The three variables included were C-source, N-source, and nitrogen-to-carbon ratio, each one at three levels, as required by the design (**Table 2**). The same design and cultures were used to gather data to fit response surfaces for cell dry weight, PHB concentration and PHB content (as % cell dry weight). A description of the design space and parameter coding can be found in the Supplementary Materials (Supplementary Figure S4).

**Table 2:** Factors and levels used in the response surface methodology experiments.

Factors investigated	Design levels
Carbon source (molar basis)	100% methane
	50% methane, 50% methanol
	100% methanol
Nitrogen source (molar basis)	100% ammonium
	50% ammonium, 50% nitrate
	100% nitrate
Nitrogen-to-Carbon ratio (molar basis)	0.005
	0.025
	0.045


## Results

### Effect of Carbon and Nitrogen Source and Inoculum History on Growth of *M. trichosporium* OB3b

Four factors were selected to investigate their impact on the growth of *M. trichosporium* OB3b: carbon source (methane or methanol), nitrogen source (ammonium or nitrate), nitrogen-to-carbon ratio (low and high), and inoculum history (fresh or aged, from methane-grown or methanol-grown cultures with either N-source). Growth curves obtained for all 64 cultures and 24 treatments are shown in Supplementary Figures S1–S3. The final OD_540_ values and duration of lag phases from all experiments are reported in **Tables 3, 4**, respectively.

**FIGURE 1:**
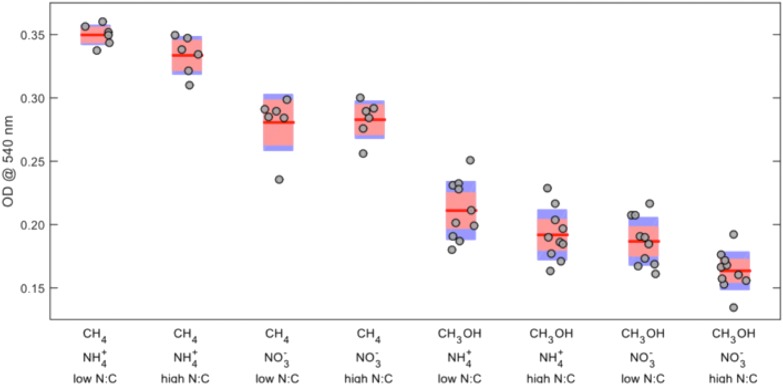
Effects of combined carbon source, nitrogen source, and nitrogen-to-carbon ratio on final OD_540_ of *Methylosinus trichosporium* OB3b. The *red lines* represent the mean values, the *red areas* represent the mean ± 1.96 × standard error, and the *blue areas* represent the mean ± one standard deviation.

The effects of each of the four factors on biomass yield were determined by ANOVA (Supplementary Table S1). Carbon source was the most significant factor, followed by nitrogen source (**Figure 1**). Overall, the average OD_540_ for methane- versus methanol-grown cells was 0.31 and 0.19, respectively, and higher yields were generally achieved using ammonium over nitrate, particularly in methane-grown cells. Except for methane-grown cells with nitrate, a low N:C ratio resulted in slight, but significant, increases in biomass. There was no significant effect of inoculum age on biomass yield except for the independent experiment using younger inocula for methanol-grown cells, in which the freshest inoculum resulted in a significantly higher biomass yield (an increase of 0.039 units).

**FIGURE 2:**
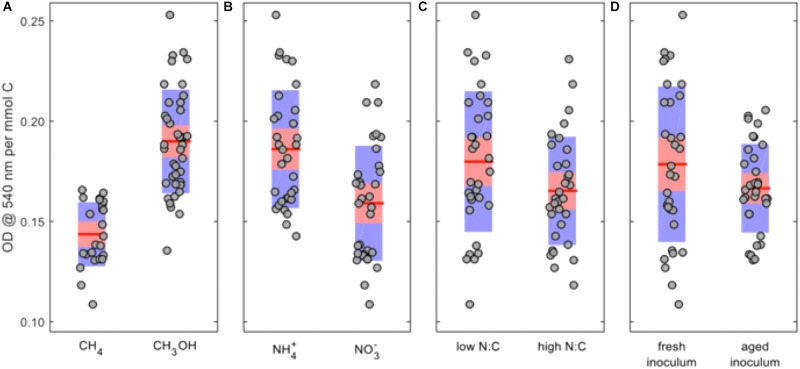
Main-factor effects on normalized OD_540_ for *Methylosinus trichosporium* OB3b cultures. **(A)** C-source; **(B)** N-source; **(C)** N:C ratio; and **(D)** inoculum history. The *red lines* represent the mean values, the *red areas* represent the mean ± 1.96 × standard error, and the *blue areas* represent the mean ± one standard deviation.

Because the absolute carbon loads were not equivalent between the methane- and methanol-grown cultures, the OD_540_ values were normalized by dividing by the moles of carbon, and the ANOVA was repeated (Supplementary Table S2). In this analysis methanol becomes the preferred C-source, ammonium remains the preferred N-source, a low N:C ratio remains favorable, and fresh inoculum was more favorable than aged inoculum (**Figure 2**).

### PHB Production as a Function of N:C Ratio

**FIGURE 3:**
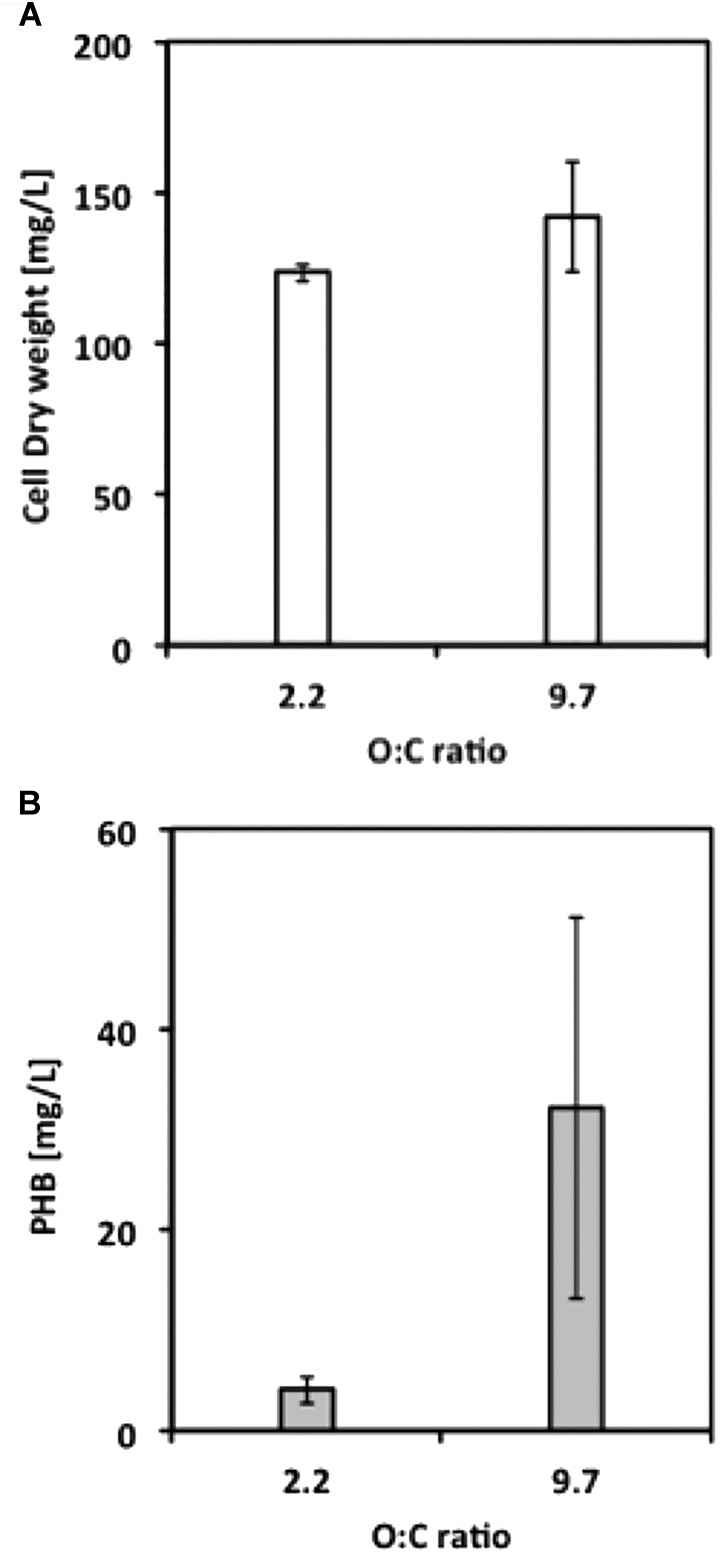
**(A)** Final cell dry weight and **(B)** PHB concentration from *M. trichosporium* OB3b cultures grown under different O:C ratios. Error bars indicate standard deviation (*n* = 3).

**FIGURE 4:**
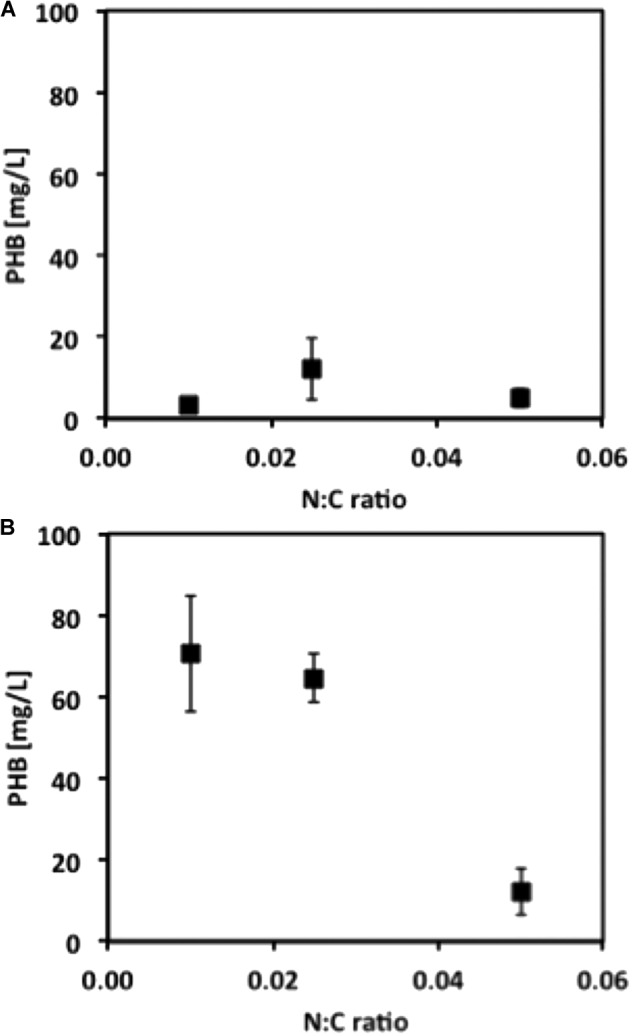
PHB concentration from *M. trichosporium* OB3b cultures grown under different N:C ratios when **(A)** methane or **(B)** methanol is used as C-source. Error bars indicate standard deviation (*n* = 3).

Our next objective was to determine optimized conditions for production of PHB by *M. trichosporium* OB3b. Cultures were grown with either methane or methanol and with ammonium or nitrate in a smaller liquid volume (50 mL) and in either 250-mL or 1-L bottles, corresponding to O:C ratios of 2.2:1 and 9.7:1, respectively, to avoid oxygen limitation. Although growth on methane was equivalent for cultures in both the 250-mL and 1-L bottles, PHB production increased 10-fold for cultures in 1-L bottles, suggesting that PHB production requires a high O:C ratio (**Figure 3**). PHB content was subsequently determined as a function of the N:C ratio and cell dry weight. Nitrate resulted in higher yields of PHB in methane-grown cells, whereas ammonium was the superior N-source for PHB production in methanol-grown cells (data not shown). For both carbon sources, the optimal N:C ratio was 0.025 for maximum %PHB of total cell dry weight, although the N:C ratio of 0.01 was equally effective for PHB production in methanol-grown cells (**Figure 4**). Furthermore, PHB production was five times higher in methanolthan methane-grown cells, indicating a preference for methanol for PHB production.

### Growth Response Surface Analysis

**FIGURE 5:**
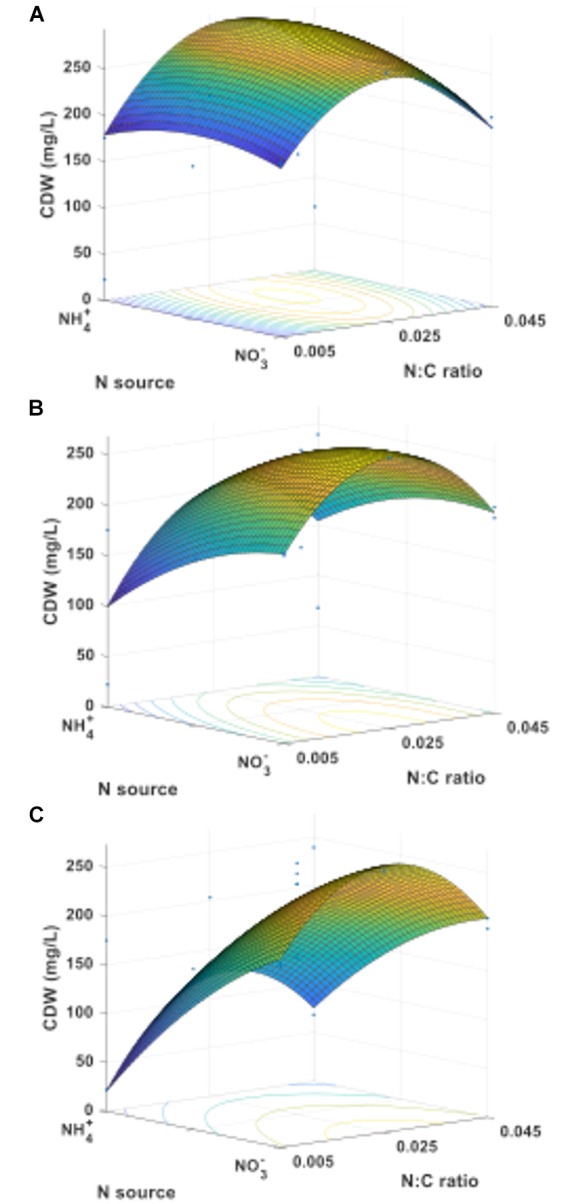
Cell dry weight response surfaces for *M. trichosporium* OB3b growing on **(A)** methane, **(B)** an equimolar mixture of methane and methanol, or **(C)** methanol as C-source.

Based on the results obtained in the statistical regression analysis, a face-centered central composite design with three factors: carbon source, nitrogen source, and nitrogen-to-carbon ratio, at three levels each, was selected for the RSM analysis, for a total of 15 treatments. Cultures were grown under oxygen-rich conditions (O:C ratio of 9.7). **Figure 5** shows the response surfaces for final cell dry weight when methane (**Figure 5A**), an equimolar mixture of methane and methanol (**Figure 5B**), or methanol (**Figure 5C**) were used as carbon source and N-sources were either combined or pure. First, it is interesting to note the conditions where the maxima occur: (a) for methane, the N-source is mixed and the N:C ratio = 0.028, (b) for methane plus methanol, the N-source is nitrate and the N:C ratio = 0.026, and (c) for methanol, the N-source is nitrate and the N:C ratio = 0.026. It is also important to note the different shapes of the response surfaces, denoting different susceptibilities to the factors tested depending on the carbon source used. For example, the N-source had a much stronger impact on biomass accumulation in methanol-grown cultures compared to cultures grown on a methane alone or in a mixture of methane and methanol.

The complete array of response surfaces obtained for growth (constant C-sources, constant N-sources, and constant N:C ratios) can be found in Supplementary Figure S5. An interesting finding from these results is the strong dependence of cell dry weight on the provided C-source when ammonium is the N-source (Supplementary Figure S5d) compared to a relative independence on C-source when nitrate is the N-source (Supplementary Figure S5f).

### PHB Concentration Response Surface Analysis

**FIGURE 6:**
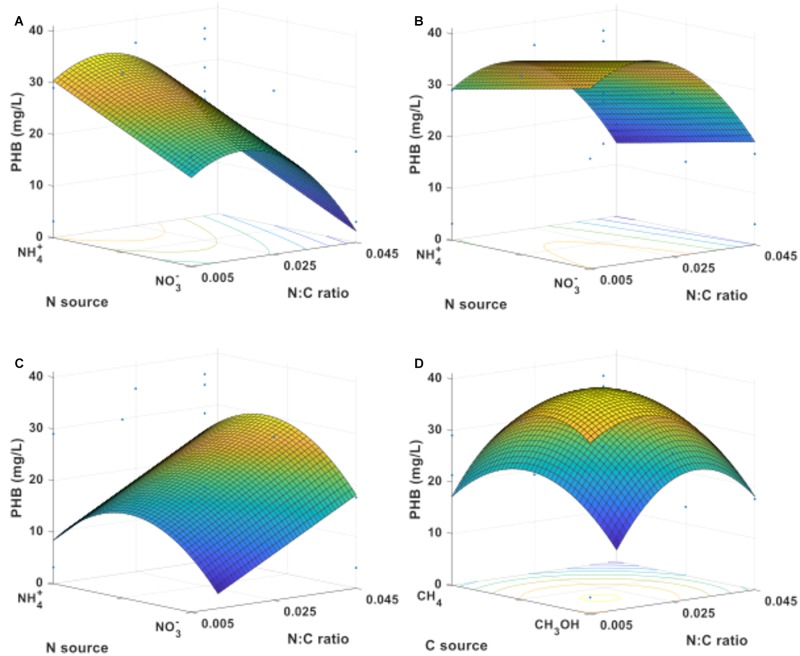
PHB concentration response surfaces for *M. trichosporium* OB3b growing on **(A)** methane, **(B)** an equimolar mixture of methane and methanol, or **(C)** methanol as C-source. **(D)** Response surface leading to optimal concentration (nitrate as N-source).

The same treatments were used to investigate the resulting PHB concentration in *M. trichosporium* OB3b as a function of C-source, N-source, and N:C ratios. Samples for PHB quantification were analyzed as soon as the optical density of the cultures reached or exceeded 0.25 and following at least 24 h of cultivation. **Figure 6** shows the response surface for the PHB concentration obtained from cultures growing on methane (**Figure 6A**), an equimolar mixture of methane and methanol (**Figure 6B**), or methanol (**Figure 6C**) with ammonium or nitrate as N-source. For methane-grown cells, the maximum PHB concentration (34 mg/L) occurred at the boundary of the experimental region representing ammonium as the sole nitrogen source (**Figure 6A**). When changing the carbon source to methanol (**Figure 6C**), the position of the maximum (37 mg/L) moved to the nitrate boundary of the experimental region. The use of mixtures of methane and methanol as carbon source had synergistic effects and higher amounts of PHB were produced than when either carbon source was used alone; the maximum PHB concentration was 39 mg/L with nitrate as the sole N-source (**Figure 6B**). In every case, the maximum value occurred at a low nitrogen-to-carbon ratio of 0.017. The complete set of response surfaces for the PHB concentration can be found in Supplementary Figure S6. As before, the shape and ranges of the surfaces help assess the dependence of PHB concentration on the various factors and conditions tested.

Based on the data, maximum PHB production was predicted to occur when the carbon source was composed of 30% methane and 70% methanol, using nitrate as the sole nitrogen source, and at a nitrogen-to-carbon ratio of 0.017 (**Figure 6D**). The predictions from the model were verified experimentally by quintuplicate cultures in two blocks at conditions close to the predicted maximum. The observed value of the PHB concentration (48.7 ± 8.3 mg/L) fell within the 95% confidence limit of the model prediction. This corresponded to a PHB cell content of 52.5 ± 6.3% on a cell dry weight basis.

## Discussion

Although previous studies have been performed to optimize cultivation conditions for PHB production in *M. trichosporium* OB3b (Rostkowski et al., 2013; Zhang et al., 2017), the present work is novel in (1) presenting interactions across multiple variables using statistical regression analysis and RSM models, and (2) showing that, under appropriate conditions, significant PHB production can take place during growth.

We initially examined the growth and production of PHB by *M. trichosporium* OB3b when varying the carbon source, nitrogen source, nitrogen-to-carbon ratio, and inoculum history. These experiments were used to determine the significance of each variable and of their respective interactions, based on regression analyses (*p*-value < 0.05; Supplementary Tables S1, S2). Although most of the parameters significantly affecting growth and growth normalized by the moles of supplied carbon are the same, the history of the inoculum and the interaction between the carbon source and N:C ratio have more significance when looking at the latter. From these analyses we can deduce models (Supplementary Equations S1, S2) that help predict the levels of biomass expected under a given set of growth conditions. It is important to note that these models provide useful information within the experimental space tested and should not be used for extrapolated conditions. It is also important to note that because the C-sources and N-sources tested lead to different physiologies and metabolic responses, within the context of these models, these factors can only be used in a discrete context (either methane or methanol; either ammonium or nitrate). Being conscious of these constraints, valuable information can be extracted from the models.

Among the variables tested, the greatest biomass was achieved when using methane over methanol as carbon source and ammonium over nitrate as nitrogen source. Methanol was modeled as a more productive carbon source when biomass accumulation (as measured by OD_540_; **Table 3**) was corrected for total moles available carbon due to the low mass-transfer of methane to the medium. Inoculum age was not a significant factor in biomass accumulation, but older inocula resulted in significantly longer lag phases when methane was used as C-source (**Table 4**).

**Table 3:** Final OD_540_ for *Methylosinus trichosporium* OB3b growth experiments.

C-source	N-source	N:C ratio	CH_4_-grown inoculum		CH_3_OH-grown inoculum	
			Fresh	Aged	Fresh	Aged
CH_4_	NH_4_^+^	Low	0.346 @ 0.009	0.354 @ 0.006	–	–
CH_4_	NH_4_^+^	High	0.336 @ 0.013	0.331 @ 0.020	–	–
CH_4_	NO_3_^-^	Low	0.270 @ 0.030	0.291 @ 0.007	–	–
CH_4_	NO_3_^-^	High	0.275 @ 0.018	0.291 @ 0.008	–	–
CH_3_OH	NH_4_^+^	Low	0.225 @ 0.012	0.193 @ 0.007	0.240 @ 0.016	0.190 @ 0.014
CH_3_OH	NH_4_^+^	High	0.183 @ 0.010	0.193 @ 0.014	0.223 @ 0.009	0.174 @ 0.015
CH_3_OH	NO_3_^-^	Low	0.197 @ 0.017	0.173 @ 0.016	0.208 @ 0.000	0.170 @ 0.005
CH_3_OH	NO_3_^-^	High	0.156 @ 0.021	0.160 @ 0.007	0.182 @ 0.014	0.163 @ 0.007


**Table 4:** Lag phase (days) for *M. trichosporium* OB3b growth experiments.

C-source	N-source	N:C ratio	CH_4_-grown inoculum		CH_3_OH-grown inoculum	
			Fresh	Aged	Fresh	Aged
CH_4_	NH_4_^+^	Low	1	2.5	–	–
CH_4_	NH_4_^+^	High	1.5	2.5	–	–
CH_4_	NO_3_^-^	Low	1.5	2.5	–	–
CH_4_	NO_3_^-^	High	1.5	2.5	–	–
CH_3_OH	NH_4_^+^	Low	12–14	9	5–5.5	9.5–10.5
CH_3_OH	NH_4_^+^	High	10–13	12–13	5.5	9–11
CH_3_OH	NO_3_^-^	Low	12–14	10.5	4.5–5	9–11.5
CH_3_OH	NO_3_^-^	High	10.5–11	9	4.5	8.5


Oxygen has been established as an important factor affecting the production of PHB. Rostkowski et al. (2013) used substrate partitioning parameters (between biomass and PHB) for nitrate and ammonium which establishes the oxygen-to-carbon stoichiometric ratio necessary for PHB production at approximately 1.50. In fact, the O:C ratio was indeed a critical factor at least for methane-grown cells, suggesting that O_2_ limitation is disadvantageous for PHB accumulation by *M. trichosporium* OB3b. However, although PHB production was observed at an O:C ratio of 2.2, the production was significantly improved when the ratio was raised to 9.7 (**Figure 3**). It is important to note that (1) the difference in O:C ratio did not affect the level of biomass produced, and that, (2) in this study, the PHB production took place during growth and was assessed at the onset of stationary phase (and not under starvation conditions as performed in most studies), which may explain the difference in optimal O:C ratio.

This information, along with the effective range of N:C ratio leading to PHB production without negatively impacting growth (**Figure 4**), was used to determine the conditions and define the experimental space for RSM. The analysis of the response surfaces obtained for biomass allow us to better understand the interactions between the factors tested (C-source, N-source, and N:C ratio), determine how they impact the responses investigated (biomass and PHB yields) and establish the extent of the variations as the parameters are changed. The models resulting from these analyses can also be used to determine the conditions leading to the optimal response within the experimental space. For example, from the RSM model, the maximum biomass yield was predicted when *M. trichosporium* OB3b was grown on methane and a mixture of 64% ammonium and 36% nitrate with a N:C ratio of approximately 0.028 (Supplementary Figure S5a). However, a more stable surface with high biomass yield was observed for a large set of conditions when the N:C ratio was kept at 0.025 and the N-source was composed mostly of nitrate, regardless of the C-source used (Supplementary Figures S5f,h).

When looking at PHB production as the response of interest, methane-grown cells accumulated more PHB with ammonium as the nitrogen source (**Figure 6A**), whereas nitrate was preferred for PHB accumulation in methanol-grown cultures (**Figure 6C**). Interestingly, a greater amount of PHB accumulated when using a mixture of methane and methanol than when using either carbon source alone (**Figure 6B**), indicating a synergistic effect between the two carbon sources. In these cultures, nitrate was the preferred N-source for PHB accumulation. The maximum accumulation of PHB was predicted and confirmed experimentally from a mixture of 30% mol methane and 70% mol methanol as C-source, nitrate as the sole N-source, and a N:C ratio of 0.017. Many important observations can be made from these results. (1) The optimal N:C ratio for PHB production (0.017) is lower than the optimum ratio for biomass accumulation (0.025–0.028), highlighting how different conditions are required to optimize growth and PHB production during growth. (2) The maximum in PHB production (48.7 ± 8.3 mg/L) was obtained in simple batch operation and could be further improved through fed-batch operation. (3) The resulting PHB cell content of 52.5 ± 6.3% on a cell dry weight basis is significantly higher than that obtained in typical batch cultures without any period of nitrogen limitation. In fact, unlike prior studies on optimizing PHB accumulation in *M. trichosporium* OB3b, the results were obtained without relying on extended periods of nutrient limitation (Asenjo and Suk, 1986; Doronina et al., 2008; Rostkowski et al., 2013; Zhang et al., 2017). This suggests that PHB production occurs when the bacterial population is actively growing, albeit while maintaining a low N:C ratio and a high O:C ratio in the cultures.

In addition to finding optimal conditions for growth and PHB production, the investigation of response surfaces enabled determination of conditions for which output (cells or PHB) was less affected by varying conditions. For example, the PHB concentration showed less overall dependence on N-source when cells were grown on equimolar mixtures of methane and methanol than with single carbon sources.

Taking these results together, this study highlights how PHB production by *M. trichosporium* OB3b can be maximized by growth on a methane–methanol mixture as the C-source, nitrate for the N-source, and a two-phase N:C ratio, in which a higher N:C ratio would favor biomass accumulation and a lower N:C ratio would benefit PHB accumulation. Proper aeration and a high O:C ratio is a critical factor for PHB production in *M. trichosporium* OB3b.

By determining the impact of multiple factors, this study serves as a guideline for establishing optimized industrial conditions for PHB production by methanotrophic bacteria using different C1 compounds and combinations as feedstock. In addition, this modeling exercise helped define specific conditions to maximize either biomass or PHB production and reveal the most critical conditions to control biological processes. As more commercially relevant products resulting from the bioconversion of methane are being identified or developed through genetic engineering, and as more methanotrophic bacterial strains undergo industrialization, it becomes important to develop rapid and efficient strategies facilitating and optimizing bioprocessing. The present study demonstrates how RSM can help rapidly identify optimal conditions for production, even in well-known systems.

## Author Contributions

JZC, LS, and DS conceived the idea and wrote the manuscript. JZC carried out the experiments. JZC and DS created the images and plots. LS and DS supervised the work. All authors have given consent to the final version of the manuscript.

## Conflict of Interest Statement

The authors declare that the research was conducted in the absence of any commercial or financial relationships that could be construed as a potential conflict of interest.

## Footnotes

1 https://www.mathworks.com/matlabcentral/fileexchange/26508-notboxplot?requestedDomain=true
