# Supplementary material for: Defining Nutrient Combinations for Optimal Growth and Polyhydroxybutyrate Production by Methylosinus trichosporium OB3b Using Response Surface Methodology
Source: Front Microbiol. 2018 Jul 18;9:1513. doi: 10.3389/fmicb.2018.01513 (PMC6058025; doi:10.3389/fmicb.2018.01513)
Supplement: Supplementary file 1 [file Data_Sheet_1.pdf]

Supplementary Materials

Defining nutrient combinations for optimal growth and polyhydroxybutyrate production by *Methylosinus trichosporium* OB3b using Response Surface Methodology

Jorge A. Zaldívar Carrillo[1], Lisa Y. Stein[2], Dominic Sauvageau[1#]

[1] Department of Chemical and Materials Engineering, University of Alberta, Edmonton, AB, T6G 1H9, Canada
[2] Department of Biological Sciences, University of Alberta, Edmonton, AB, T6G 2V2, Canada

[#]Corresponding author:
Email: dominic.sauvageau@ualberta.ca
Phone: +1 780 492 8092
Fax: +1 780 492 2881

Effect of carbon and nitrogen source and inoculum history on growth of *M. trichosporium* OB3b – Growth Curves

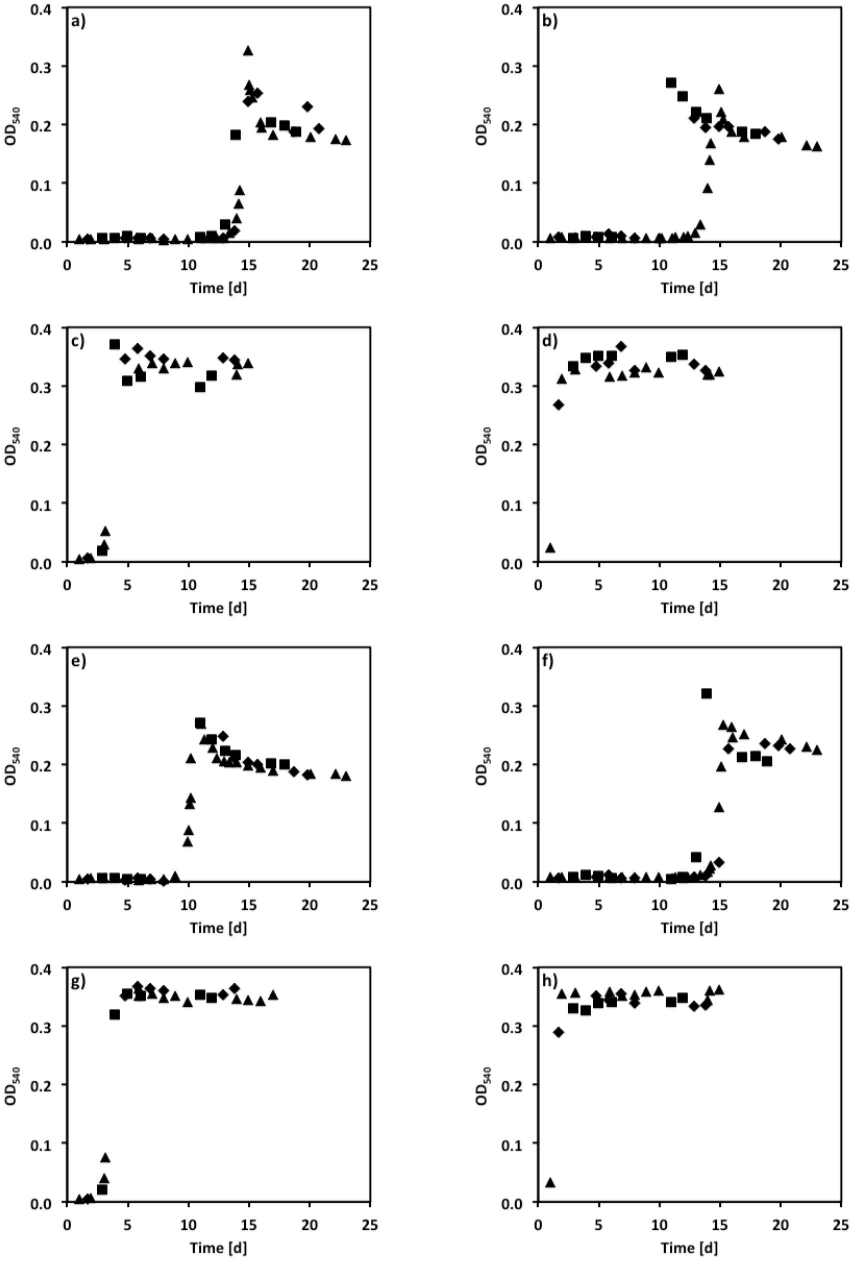

Fig. S1. Growth curves of *M. trichosporium* OB3b for treatments (methane-grown inocula, ammonium as nitrogen source) detailed in Table 1 for the second-order statistical regression analysis. Conditions: a) methanol, high N:C, aged inoculum; b) methanol, high N:C, fresh inoculum; c) methane, high N:C, aged inoculum; d) methane, high N:C, fresh inoculum; e) methanol, low N:C, aged inoculum; f) methanol, low N:C, fresh inoculum; g) methane, low N:C, aged inoculum; h) methane, low N:C, fresh inoculum.

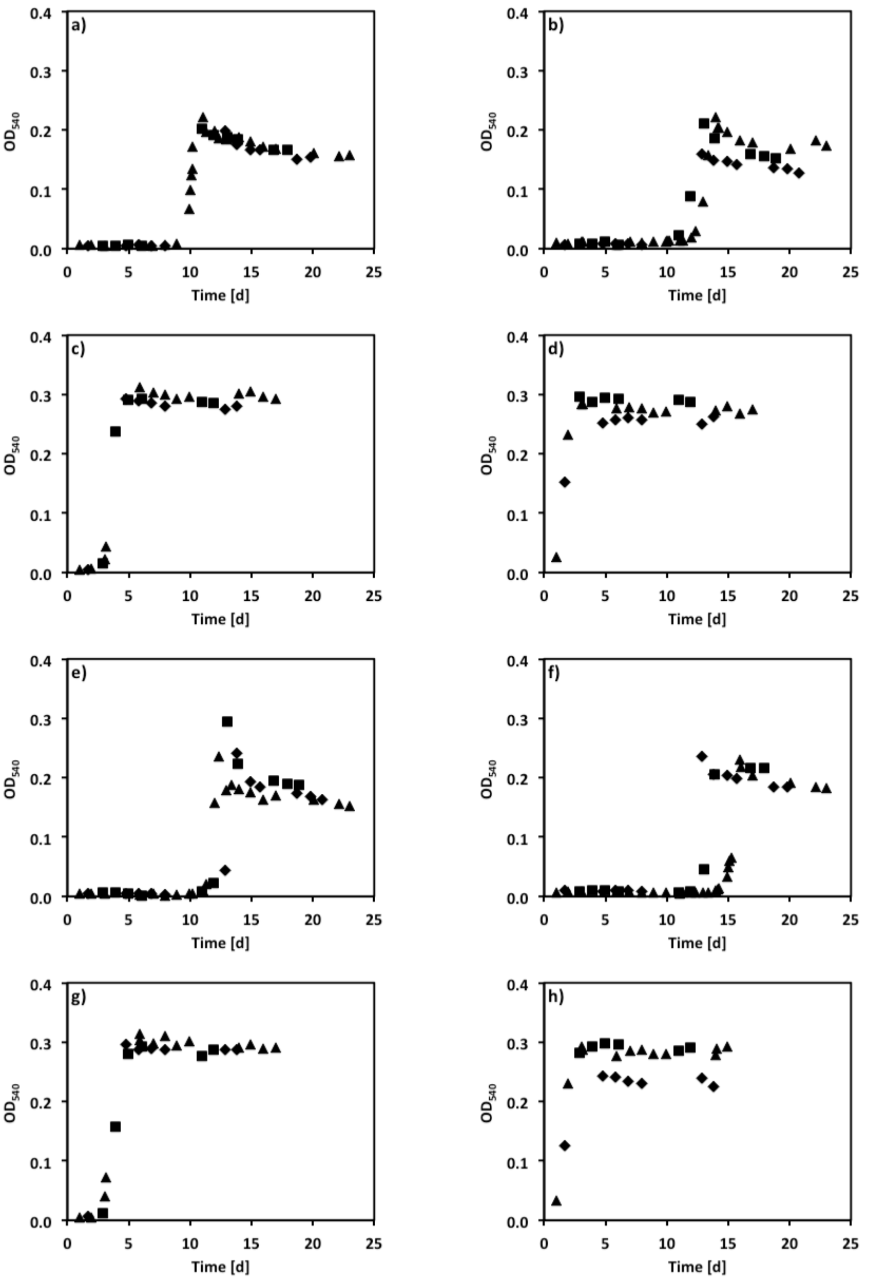

Fig. S2. Growth curves of *M. trichosporium* OB3b for treatments (methane-grown inocula, nitrate as nitrogen source) detailed in Table 1 for the second-order statistical regression analysis. Conditions: a) methanol, high N:C, aged inoculum; b) methanol, high N:C, fresh inoculum; c) methane, high N:C, aged inoculum; d) methane, high N:C, fresh inoculum; e) methanol, low N:C, aged inoculum; f) methanol, low N:C, fresh inoculum; g) methane, low N:C, aged inoculum; h) methane, low N:C, fresh inoculum.

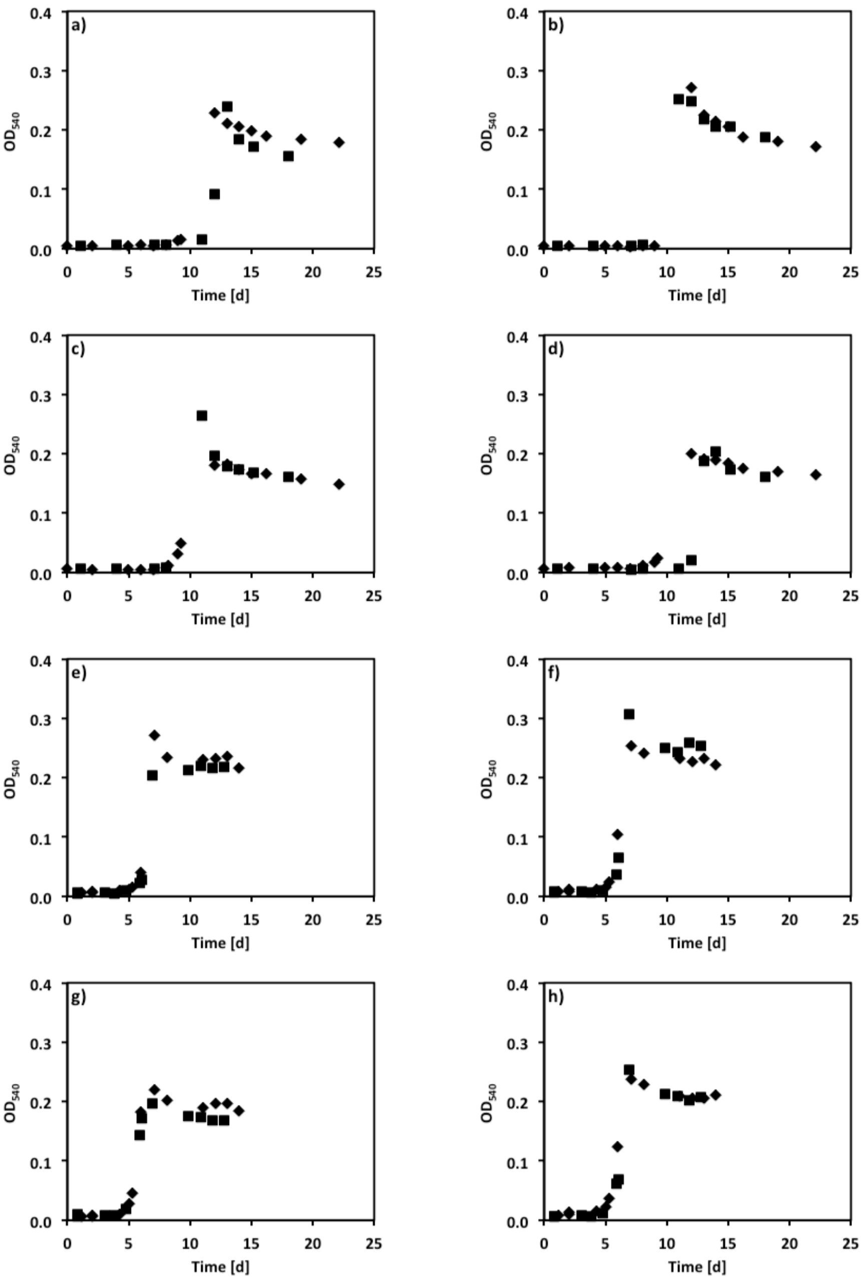

Fig. S3. Growth curves of *M. trichosporium* OB3b for treatments (methanol-grown inocula) detailed in Table 1 for the second-order statistical regression analysis. Conditions: a) methanol, ammonium, high N:C, aged inoculum; b) methanol, ammonium, low N:C, aged inoculum; c) methanol, nitrate, high N:C, aged inoculum; d) methanol, nitrate, low N:C, aged inoculum; e) methanol, ammonium, high N:C, fresh inoculum; f) methanol, ammonium, low N:C, fresh inoculum; g) methanol, nitrate, high N:C, fresh inoculum; h) methanol, nitrate, low N:C, fresh inoculum.

Effect carbon and nitrogen source and inoculum history on growth of *M. trichosporium* OB3b – Analysis of Variance

Table S1: Analysis of variance for factor effects on growth experiments. The response factor is OD_{540}, as a measure of biomass concentration. Significant effects are highlighted in bold red.

**Source**	**Sum of squares[(1)]**	**Degrees of freedom**	**Mean Square**	**F**	**p-value**
**C-source**	**0.22822**	**1**	**0.22822**	**950.07**	**9.66×10^{-34}**
**N-source**	**0.02794**	**1**	**0.02794**	**116.30**	**1.55×10^{-14}**
**N:C ratio**	**0.00299**	**1**	**0.00299**	**12.44**	**9.23×10^{-4}**
Inoculum	0.00052	1	0.00052	2.15	1.49×10^{-1}
**C-source • N-source**	**0.00423**	**1**	**0.00423**	**17.60**	**1.14×10^{-4}**
C-source • N:C ratio	0.00076	1	0.00076	3.17	8.13×10^{-2}
**C-source • Inoculum**	**0.00380**	**1**	**0.00380**	**15.82**	**2.29×10^{-4}**
N-source • N:C ratio	0.00019	1	0.00019	0.80	3.75×10^{-1}
N-source • Inoculum	0.00063	1	0.00063	2.64	1.11×10^{-1}
N:C ratio • Inoculum	0.00025	1	0.00025	1.06	3.09×10^{-1}
C-source • N-source • N:C ratio	0.00047	1	0.00047	1.96	1.68×10^{-1}
C-source • N-source • Inoculum	0.00006	1	0.00006	0.23	6.30×10^{-1}
**C-source • N:C ratio • Inoculum**	**0.00103**	**1**	**0.00103**	**4.29**	**4.37×10^{-2}**
N-source • N:C ratio • Inoculum	0.00001	1	0.00001	0.02	8.80×10^{-1}
Error	0.01177	49	0.00024		
Total	0.28149	63			

[(1)] Constrained (Type III) sums of squares.

Based on the results obtained in Table S1, the resulting model for biomass produced ($\hat{y}$), as measured by OD_{540}, as a function of the parameters investigated is:

$$\hat{y} = \alpha_1 x_1 + \alpha_2 x_2 + \alpha_3 x_3 + \alpha_{12} x_1 x_2 + \alpha_{14} x_1 x_4 + \alpha_{134} x_1 x_3 x_4 \qquad \text{(S1)}$$

In the equation, x_i represents the factors investigated (1: C-source, 2: N-source, 3: N:C ratio, 4: inoculum history), α_i represents the corresponding coefficients, and α_{ij} and α_{ijk} represent the coefficients for interactions.

Table S2: Analysis of variance for factor effects on growth experiments. The response factor is normalized OD_{540} per mol of carbon substrate, as a measure of biomass yield. Significant effects are highlighted in bold red.

Source	Sum of squares[(1)]	Degrees of freedom	Mean Square	F	p-value
**C-source**	**0.03217**	**1**	**0.03217**	**182.07**	**4.00×10^{-18}**
**N-source**	**0.01102**	**1**	**0.01102**	**62.40**	**2.72×10^{-10}**
**N:C ratio**	**0.00227**	**1**	**0.00227**	**12.89**	**7.63×10^{-4}**
**Inoculum**	**0.00113**	**1**	**0.00113**	**6.39**	**1.48×10^{-2}**
C-source • N-source	0.00000	1	0.00000	0.02	8.81×10^{-1}
**C-source • N:C ratio**	**0.00124**	**1**	**0.00124**	**7.03**	**1.08×10^{-2}**
**C-source • Inoculum**	**0.00266**	**1**	**0.00266**	**15.03**	**3.16×10^{-4}**
N-source • N:C ratio	0.00002	1	0.00002	0.10	7.51×10^{-1}
N-source • Inoculum	0.00027	1	0.00027	1.53	2.22×10^{-1}
N:C ratio • Inoculum	0.00042	1	0.00042	2.38	1.29×10^{-1}
C-source • N-source • N:C ratio	0.00015	1	0.00015	0.83	3.66×10^{-1}
C-source • N-source • Inoculum	0.00000	1	0.00000	0.01	9.18×10^{-1}
**C-source • N:C ratio • Inoculum**	**0.00078**	**1**	**0.00078**	**4.42**	**4.07×10^{-2}**
N-source • N:C ratio • Inoculum	0.00000	1	0.00000	0.00	9.64×10^{-1}
Error	0.00866	49	0.00018		
Total	0.06412	63			

[(1)] Constrained (Type III) sums of squares.

Based on the results obtained in Table S2, the resulting model for biomass produced per mole of carbon substrate ($\hat{y}^*$), as measured by OD_{540} per mol of carbon substrate, as a function of the parameters investigated is:

$$\hat{y}^* = \alpha_1^* x_1 + \alpha_2^* x_2 + \alpha_3^* x_3 + \alpha_4^* x_4 + \alpha_{13}^* x_1 x_3 + \alpha_{14}^* x_1 x_4 + \alpha_{134}^* x_1 x_3 x_4 \tag{S2}$$

In the equation, x_i represents the factors investigated (1: C-source, 2: N-source, 3: N:C ratio, 4: inoculum history), α_i^* represents the corresponding coefficients, and α_{ij}^* and α_{ijk}^* represent the coefficients for interactions.

Response Surface Methodology (RSM) – Experimental Space and Parameter Coding

The design space forms a cubic three-dimensional region where the axes correspond to the carbon source (x_1), ranging from pure methane to pure methanol, with mixtures between them; the nitrogen source (x_2), ranging from pure ammonium to pure nitrate, with mixtures of both along the axis, and the nitrogen-to-carbon ratio (x_3), ranging from 0.005 to 0.045. The experimental space is depicted in Fig. S4.

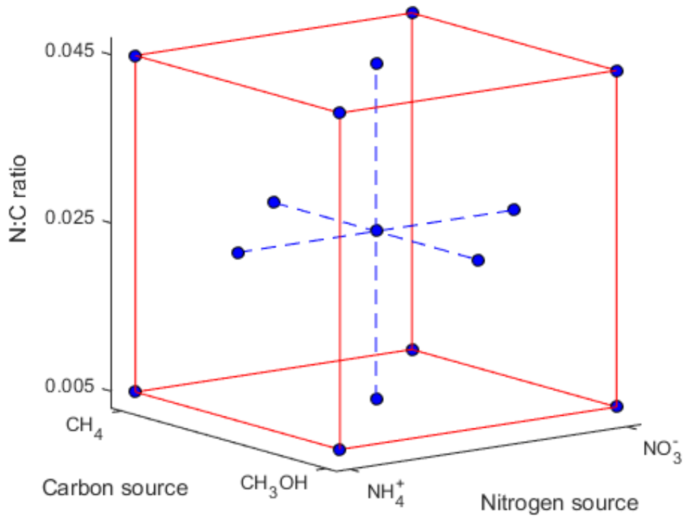

Fig. S4: Experimental space points for the face-centered central composite design. Three levels of each variable were used in the experiment. Carbon source: pure methane, pure methanol and an equimolar mixture. Nitrogen source: pure ammonium, pure nitrate and an equimolar mixture. Nitrogen-to-carbon ratio: 0.005, 0.025, and 0.045. Four replicates were run at the center point.

To represent the carbon source, variable x_1 was used, coded as follows: let c_1 and c_2 be the molar fraction of methane and methanol in the carbon source. The coded variable for carbon source was:

$$x_1 = c_2 - c_1 \quad \text{(S3)}$$

To represent the nitrogen source, variable x_2 was used, coded as follows: let n_1 and n_2 be the molar fraction of ammonium and nitrate in the nitrogen source. The coded variable for nitrogen source was:

$$x_2 = n_2 - n_1 \quad \text{(S4)}$$

To represent the nitrogen-to-carbon ratio, variable x_3 was used, coded as follows: let r_1 and r_2 be the lower and upper limits of the nitrogen-to-carbon ratio for the experiment. If r is the nitrogen-to- carbon ratio, the coded variable for nitrogen-to-carbon ratio was:

$$x_3 = \frac{r - \frac{r_1 + r_2}{2}}{\frac{r_2 - r_1}{2}} \qquad \text{(S5)}$$

As per the face-centered central composite design, coded values of -1, 0 and 1 were used for all the independent variables.

This design allows the fitting of a second order polynomial of the form:

$$\hat{y} = b_o + b_1 x_1 + b_2 x_2 + b_3 x_3 + b_{11} x_1^2 + b_{22} x_2^2 + b_{33} x_3^2 + b_{12} x_1 x_2 + b_{13} x_1 x_3 + b_{23} x_2 x_3 \qquad \text{(S6)}$$

Response Surfaces for Cell Dry Weight and PHB Concentration

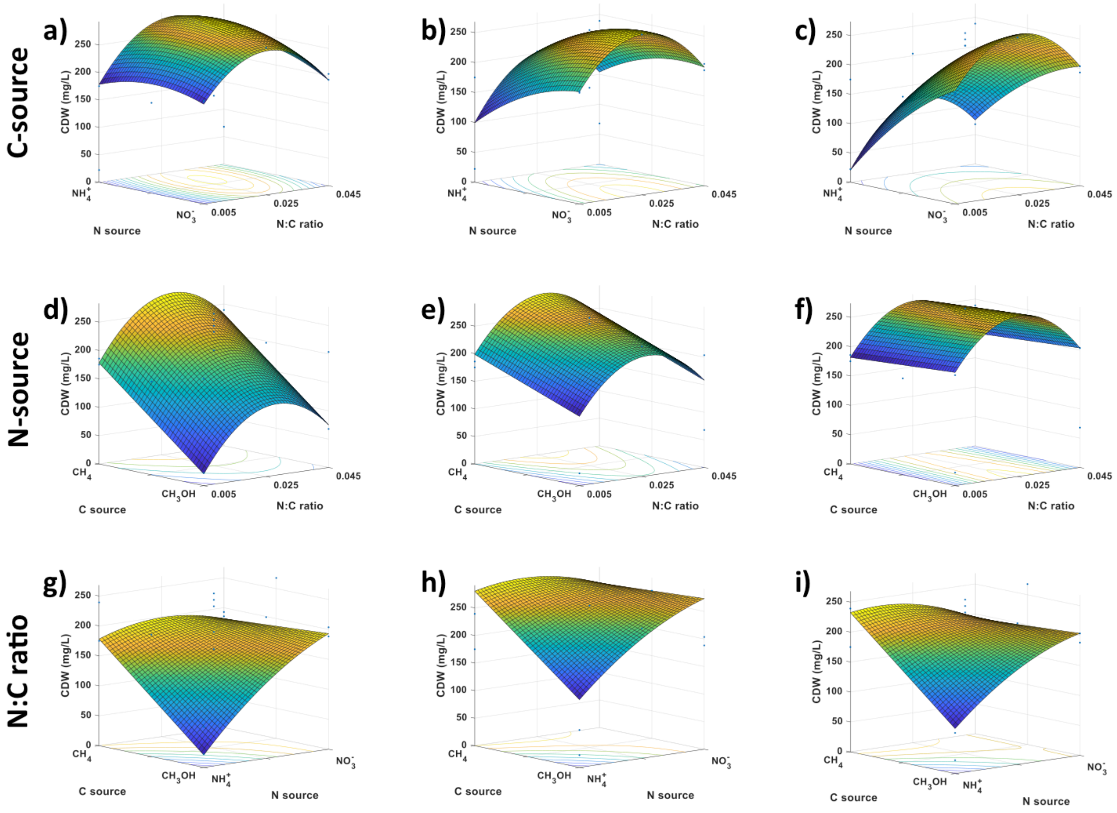

Fig. S5. Cell dry weight response surfaces for *M. trichosporium* OB3b growing on combinations of C-source, N-source and N:C ratio. First row: keeping C-sources constant: a) methane, b) equimolar mixture of methane and methanol, c) methanol; second row: keeping N-sources constant: d) ammonium, e) equimolar mixture of ammonium and nitrate, f) nitrate; third row: keeping N:C ratios constant: g) 0.005, h) 0.025, i) 0.045. The first row is shown in main body of manuscript as Fig. 5 and is reproduced here for convenience.

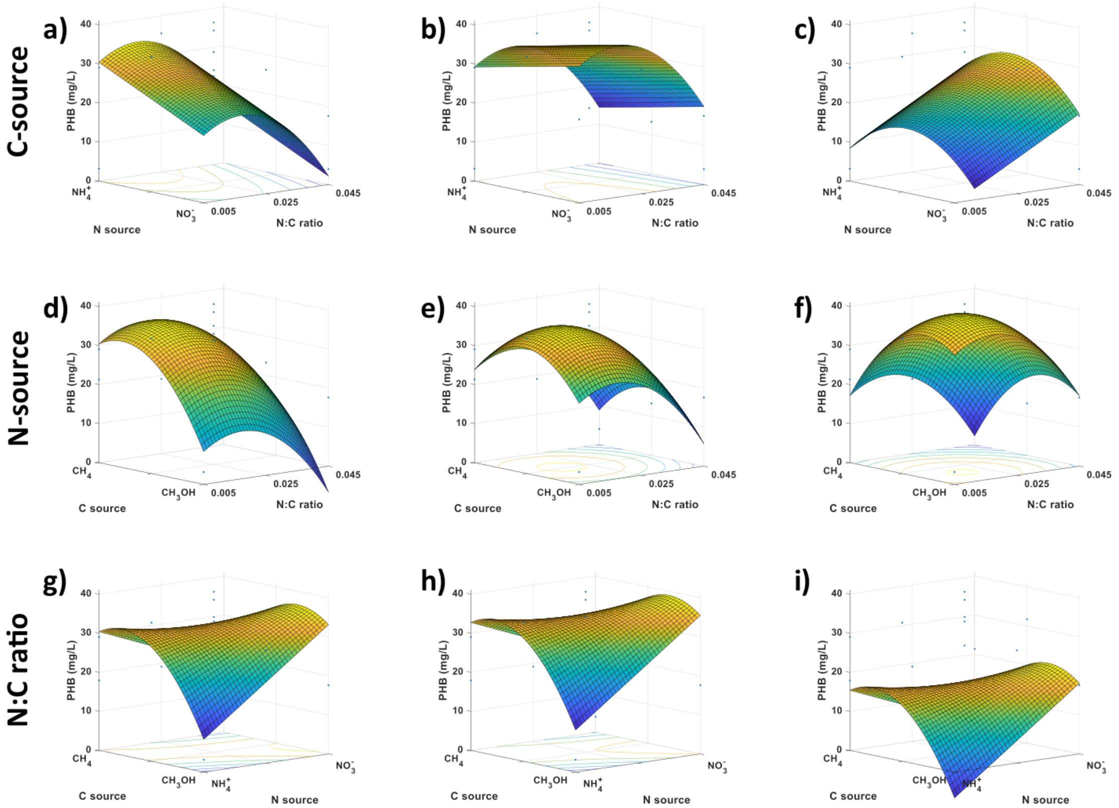

Fig. S6. PHB concentration response surfaces for *M. trichosporium* OB3b growing on combinations of C-source, N-source and N:C ratio. First row: keeping C-sources constant; second row: keeping N-sources constant; third row: keeping N:C ratios constant. The first row and Fig. 7f are shown in main body of manuscript as Fig. 6 and are reproduced here for convenience.

Example of Gas Chromatography Analysis for PHB analysis

PHB was hydrolyzed to its monomer (3-hydroxybutyric acid) and esterified with methanol according to the method described in the Materials and Methods section. Methyl 3-hydroxybutyrate was identified and compared to a standard peak of methyl benzoate (obtained from the reaction of benzoic acid with methanol in the treated samples) for quantification.

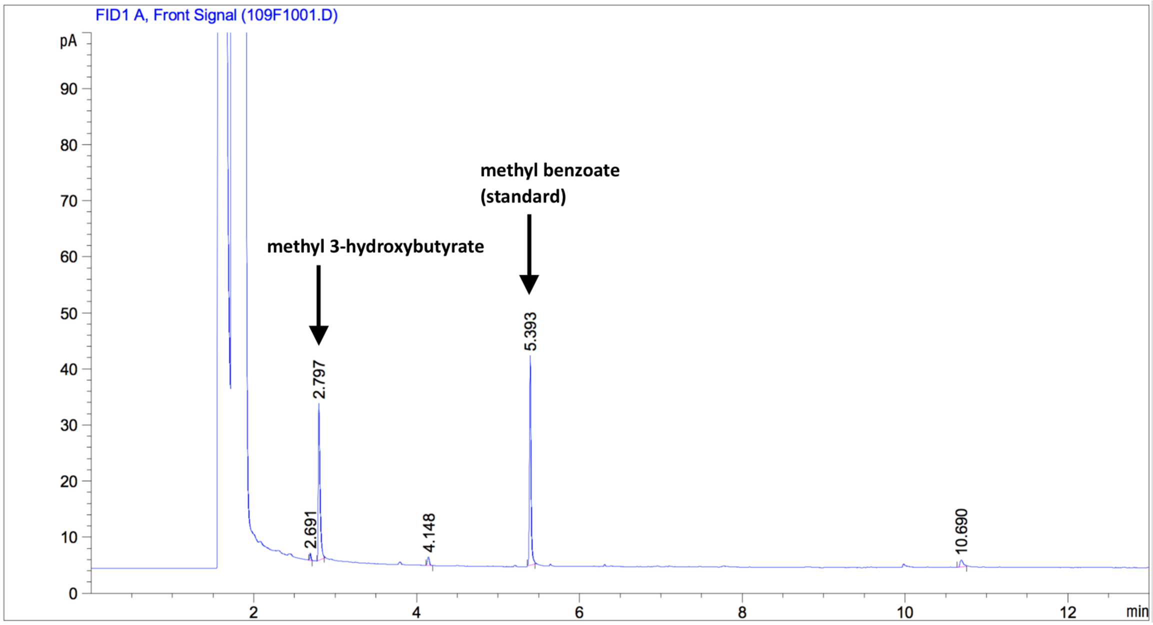

Fig. S7. Example of chromatogram for the quantification of methyl 3-hydroxybutyrate.
